# Placental Ultrasonographic Findings Due to COVID-19 Infection During Pregnancy: A Case Report

**DOI:** 10.7759/cureus.24265

**Published:** 2022-04-18

**Authors:** Athina A Samara, Antonios Koutras, Theodoros Floros, Emmanuel Kontomanolis, Sotirios Sotiriou

**Affiliations:** 1 Department of Embryology, University of Thessaly, Larissa, GRC; 2 Department of Obstetrics & Gynecology, Democritus University of Thrace, Alexandroupolis, GRC; 3 Department of Obstetrics and Gynecology, Democritus University of Thrace, Alexandroupolis, GRC

**Keywords:** sar-cov 2 infection, chorangiosis, malperfusion, pregnancy, placenta, covid-19

## Abstract

Severe acute respiratory syndrome coronavirus 2 (SARS-CoV-2) infection during pregnancy can lead to several adverse events. Here, we report a case of a 40-year-old Caucasian female, gravida 1, para 0, with a spontaneous singleton pregnancy, who presented to the emergency room during her 17th week of gestation with fever (38.8ºC), fatigue, shortness of breath, and palpitations. She tested positive for coronavirus disease 2019 (COVID-19). Ultrasonography examination revealed signs of placental involvement compatible with malperfusion, chorangiosis, deciduitis, and subchorionitis. Findings remained stable until the 20th week and gradually resolved around the 32nd week of pregnancy. A normal male neonate was delivered via elective caesarian section during the 39th week, weighing 2830 gm. The present report points toward a correlation between clinical symptomatology of COVID-19 during pregnancy and ultrasonographical features. Early detection of placental damage through the use of specific ultrasound findings could indicate which pregnancies are at increased risk for complications; however, further studies including a larger population are required to confirm these findings.

## Introduction

Severe acute respiratory syndrome coronavirus 2 (SARS-CoV-2) infection during pregnancy can lead to several adverse events such as increased rates of preterm deliveries, with rates of preterm birth at 17% compared to 8-10% reported in the general population, as well as increased rates of cesarean sections [1-3]. The developing fetus can be directly affected by viral infection through vertical transmission, or indirectly by a viral infection of the placenta [4].

In trophoblasts of infected pregnant patients, placental features such as fetal vascular malperfusion, arteriopathy and inflammation, perivillous fibrin deposition, and fetal vessel thrombosis have been reported [5]. The presence of the above-mentioned features can be indicative of the viral load and clinical severity of coronavirus disease 2019 (COVID-19) during pregnancy.

The placenta is often overlooked during routine ultrasound evaluation of a normally developing pregnancy. However, a spectrum of pathologic placental features regarding location, development, adherence, and vascularity can occur during pregnancy and sonographic evaluation of the placenta can identify abnormalities with profound implications [6-7]. Early recognition and understanding of these pathological findings can thus be critical for fetal and even maternal survival [6].

Here, we present a case of a COVID-19-positive pregnant woman during the 17th week of gestation, where the ultrasonographic placental findings were in line with the clinical symptomatology of the patient.

## Case presentation

A 40 -year-old Caucasian female, gravida 1, para 0, with a spontaneous singleton pregnancy presented to the emergency room (ER) with fever (38.8ºC), fatigue, shortness of breath, and palpitations. The patient was in her 17th week of pregnancy with a normal antenatal history. She was unvaccinated against COVID-19 and tested positive with a SARS-CoV-2 real-time polymerase chain reaction (RT-PCR) molecular test upon admission. Upon clinical examination, her saturation of peripheral oxygen (SpO2) was 95%, with normal blood pressure, a pulse of 95 per minute, and a respiratory rate of 15 breaths per minute. Furthermore, electrocardiography did not show any abnormal findings. Results of the laboratory examinations are displayed in Table 1.

**Table 1:** Laboratory examinations.

Laboratory exams/ Day	Admission Day	1^st^ Day	2^nd^ Day
White blood cells	9200/mm^3^	8500/mm^3^	7700/mm^3^
Neutrophil Ratio	71%	69%	70%
Lymphocyte Ratio	25%	27%	28%
Hematocrit	34%	33.2%	32.7%
Hemoglobin	11.4 g/dL	11.2 g/dL	10.9 g/dL
C- Reactive Protein	1.2mg/L	0.7mg/L	0.6mg/L
Erythrocyte Sedimentation Rate	46 mm/h	33mm/h	27mm/h

**Figure 1:**
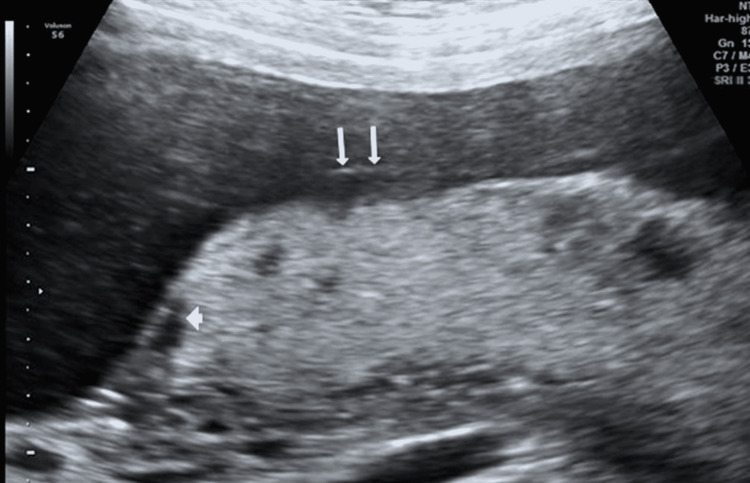
Ultrasonographic placental image at 17th week of gestation: subchorionic fibrin deposits (arrows) and blood pools (arrowhead) are observed.

Ultrasonography examination confirmed a singleton gestation, with a posterior placenta. The amniotic fluid and fetal growth were appropriate for the gestational weeks with normal fetal anatomy. During the placental evaluation, signs of placental involvement with placental lakes occupying approximately 40% of the placenta surface were recorded, as were several sites of perivillous fibrin deposit with prominent subchorionic fibrin deposition (Figure 1). These findings were compatible with malperfusion, chorangiosis, deciduitis, and subchorionitis.

The patient was treated with paracetamol for fever prevention and broad-spectrum antibiotics (cefoxitin 2g twice a day and azithromycin 400mg once a day) for the prevention of bacterial infection. She was hospitalized for two days and discharged without any further symptoms. Pregnancy follow-up with serial ultrasound scans was planned every two weeks to look for further placental complications.

**Figure 2:**
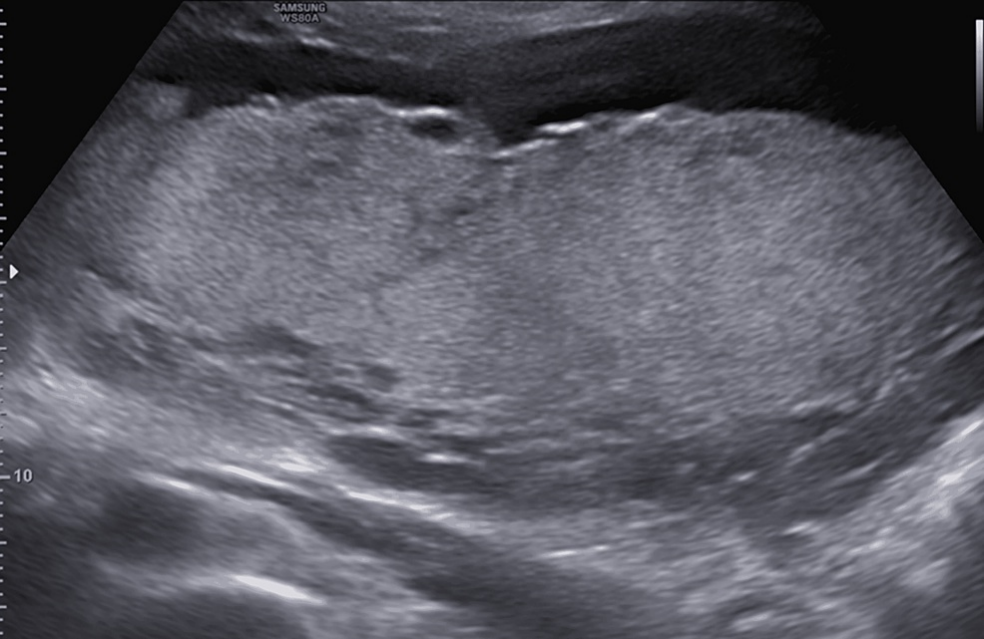
Ultrasonographic placental image at 32nd week of gestation: The pathological placental findings have been resolved.

Interestingly, the findings remained stable until the 20th week, gradually resolving around the 32nd week of pregnancy (Figure 2). Labor was planned for the 39th week of gestation via elective caesarian section upon maternal request. A normal male neonate was delivered with an APGAR (Appearance, Pulse, Grimace, Activity, and Respiration) score of 9, weighing 2830 gm.

## Discussion

The precise impact of COVID-19 on pregnancy and vice versa remains unknown. Pregnant women are more vulnerable to viral infection and they are more susceptible to developing severe illness than non-pregnant women, with an increased rate of intensive care unit admission, need for supplemental oxygen and ventilation, and mortality [8-9]. However, according to two systematic reviews, the clinical symptomatology of pregnant women infected by COVID-19 are similar to non-pregnant adults and there is no evidence that COVID-19 infected pregnant women are more likely to develop severe pneumonia or mortality [10,11].

Maternal serum pro-inflammatory and anti-inflammatory cytokine levels are tightly regulated during pregnancy [9]. The first trimester of pregnancy is skewed towards a pro-inflammatory state, whereas the last two trimesters are anti-inflammatory. Systemic pro-inflammatory cytokines such as Cxc chemokine ligand 10 (CXCL10), tumor necrosis factor-alpha (TNF-a), interleukin-18 (IL-18), interleukin-6 (IL-6), and Cxc chemokine ligand 8 (CXCL8) remain steady over the last two trimesters of pregnancy, while anti-inflammatory cytokine levels increase over time, shifting the balance away from the initial pro-inflammatory state [12]. Disruption of immune regulation with uncontrolled release of inflammatory cytokines, as seen in preeclampsia that results in systemic inflammation, places the pregnancy at risk for fetal demise and maternal death [4,12].

Viraemia may trigger a “cytokine storm” following overwhelmed maternal immune response to SARS-CoV-2 infection, with a surge in pro-inflammatory cytokines including interferon-G (IFN-G), interleukin-2 (IL-2), IL-6, interleukin-7 (IL-7), interleukin-10 (IL-10), and TNF. Others believe that increased production of inflammatory biomarkers may be triggered by placental hypoperfusion/ischemia due to maternal hypoxia following severe COVID-19 infection [13]. High levels of IL-6, indicating systemic inflammation, is characteristic of severe COVID-19 and correlates with respiratory failure. It is also a marker associated with pre-eclampsia and has been proposed as a mediator for pre-eclampsia pathogenesis [14,15].

Recently published research outcomes reported specific histopathology trophoblastic findings of positive for COVID-19 pregnancies. The above-mentioned finding include vascular malperfusion, arteriopathy, inflammation, perivillous fibrin deposition [16,17]. Furthermore, prenatal diagnosis of placentitis features using ultrasound findings as placental lakes, fibrin deposits, and subchorionic edema could serve as a useful diagnostic tool of early signs of adverse pregnancy outcomes requiring closer antenatal follow-up [18].

The present report highlights a correlation between clinical symptomatology of COVID-19 during pregnancy and ultrasonographical features. In our case, findings indicating malperfusion, chorangiosis, deciduitis and subchorionitis were presented concurrently with the beginning of clinical symptomatology of the infection, remaining stable for five weeks and resolving after a total of 12 weeks. 

## Conclusions

Placental injuries attributed to COVID-19 infection can occur even in cases with mild to moderate clinical symptomatology. Early detection of placental damage through the use of specific ultrasound findings could indicate which pregnancies are at increased risk for complications. Prior to the generalization of these findings, they must be systematically tested in a larger patient population of pregnant women. 

## References

[1] Schwartz DA (2020). An analysis of 38 pregnant women with COVID-19, Their newborn infants, and maternal-fetal transmission of SARS-CoV-2: maternal coronavirus infections and pregnancy outcomes. Arch Pathol Lab Med.

[2] Jering KS, Claggett BL, Cunningham JW, Rosenthal N, Vardeny O, Greene MF, Solomon SD (2021). Clinical characteristics and outcomes of hospitalized women giving birth with and without COVID-19. JAMA Intern Med.

[3] Bwire GM, Njiro BJ, Mwakawanga DL, Sabas D, Sunguya BF (2021). Possible vertical transmission and antibodies against SARS-CoV-2 among infants born to mothers with COVID-19: A living systematic review. J Med Virol.

[4] Moore KM, Suthar MS (2021). Comprehensive analysis of COVID-19 during pregnancy. Biochem Biophys Res Commun.

[5] Resta L, Vimercati A, Cazzato G (2021). SARS-CoV-2 and placenta: new insights and perspectives. Viruses.

[6] Rheinboldt M, Delproposto Z (2015). Sonography of placental abnormalities: a pictorial review. Emerg Radiol.

[7] Bowman ZS, Kennedy AM (2014). Sonographic appearance of the placenta. Curr Probl Diagn Radiol.

[8] Elsaddig M, Khalil A (2021). Effects of the COVID pandemic on pregnancy outcomes. Best Pract Res Clin Obstet Gynaecol.

[9] Graham C, Chooniedass R, Stefura WP (2017). In vivo immune signatures of healthy human pregnancy: Inherently inflammatory or anti-inflammatory?. PLoS One.

[10] Kasraeian M, Zare M, Vafaei H, Asadi N, Faraji A, Bazrafshan K, Roozmeh S (2022). COVID-19 pneumonia and pregnancy; a systematic review and meta-analysis. J Matern Fetal Neonatal Med.

[11] Yang Z, Wang M, Zhu Z, Liu Y (2022). Coronavirus disease 2019 (COVID-19) and pregnancy: a systematic review. J Matern Fetal Neonatal Med.

[12] Gilbert JS, Ryan MJ, LaMarca BB, Sedeek M, Murphy SR, Granger JP (2008). Pathophysiology of hypertension during preeclampsia: linking placental ischemia with endothelial dysfunction. Am J Physiol Heart Circ Physiol.

[13] Brien ME, Bouron-Dal Soglio D, Dal Soglio S (2021). Pandemic stress and SARS-CoV-2 infection are associated with pathological changes at the maternal-fetal interface. Placenta.

[14] Herold T, Jurinovic V, Arnreich C (2020). Elevated levels of IL-6 and CRP predict the need for mechanical ventilation in COVID-19. J Allergy Clin Immunol.

[15] Lockwood CJ, Yen CF, Basar M (2008). Preeclampsia-related inflammatory cytokines regulate interleukin-6 expression in human decidual cells. Am J Pathol.

[16] Wong YP, Khong TY, Tan GC (2021). The effects of COVID-19 on placenta and pregnancy: what do we know so far?. Diagnostics (Basel).

[17] Allotey J, Stallings E, Bonet M (2020). Clinical manifestations, risk factors, and maternal and perinatal outcomes of coronavirus disease 2019 in pregnancy: living systematic review and meta-analysis. BMJ.

[18] Sotiriou S, Samara AA, Tsiamalou IA (2022). Placental ultrasonographical findings during SARS-CoV-2 infection. Diagnostics.

